# Interstitial granulomatous dermatitis presenting with the rope sign in a patient with systemic lupus erythematosus

**DOI:** 10.1016/j.jdcr.2026.03.016

**Published:** 2026-03-18

**Authors:** Maya Deva, Michelle Chen, Madeline Anne Wang, Peter Bullpitt, Steven Kossard, Kevin Phan, Andrew Chen

**Affiliations:** a Department of Dermatology, Royal Prince Alfred Hospital, Sydney, Australia; b Dermatopathology, Kossard Dermatopathologists, Sydney, Australia

**Keywords:** autoimmune disease, interstitial granulomatous dermatitis, reactive granulomatous dermatitis, rheumatoid arthritis, rope sign, systemic lupus erythematosus

## Case Report

A 57-year-old man was referred to dermatology by his rheumatologist with a 2 month history of erythematous, linear indurated plaques extending vertically along his right anterior shoulder to anterior axilla, as well multiple linear plaques on his left lateral trunk. The lesions were nontender, and he denied any systemic symptoms. This was on a background of systemic lupus erythematosus diagnosed 25 years prior, which was well controlled on a stable dose of methotrexate 10 milligrams weekly.

**Fig 1:**
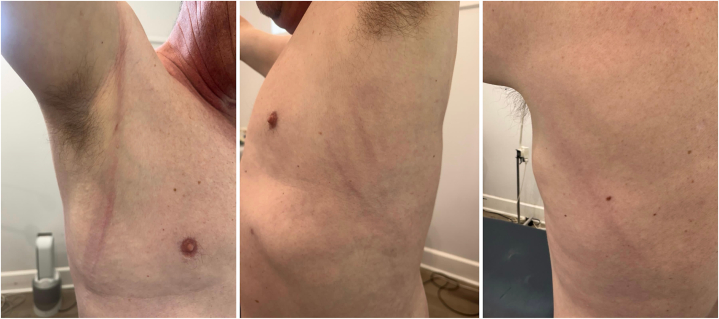
Clinical photographs of interstitial granulomatous dermatitis with rope sign demonstrating erythematous, firm, linear subcutaneous cords along the left and right axillary lines.

Examination revealed erythematous, firm, linear, indurated cords, on his right anterior shoulder extending along his anterior axillary vault, as well as multiple linear indurated cords on his far left lateral trunk and back. These were consistent clinically with the rope sign (Fig 1). Laboratory studies including full blood count, eosinophil count, and C-reactive protein were within normal limits. His most recent antinuclear antibody titer was positive at 1:640 with a speckled pattern and he had positive anti-SS-A 60, anti-Ro-52, anti-Smith, antiribonucleoprotein, and anti–double-stranded antibodies.

**Fig 2:**
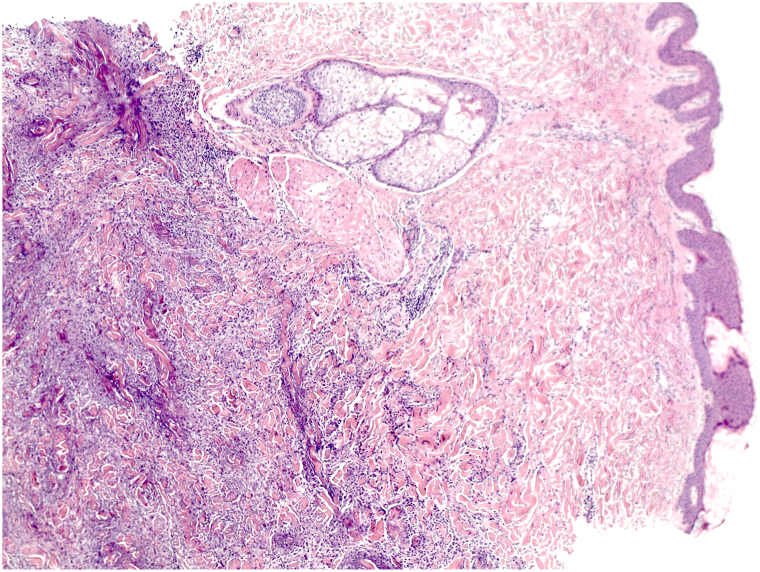
Interstitial granulomatous dermatitis skin sections through deep inflammatory cord (×50, H&E).

**Fig 3:**
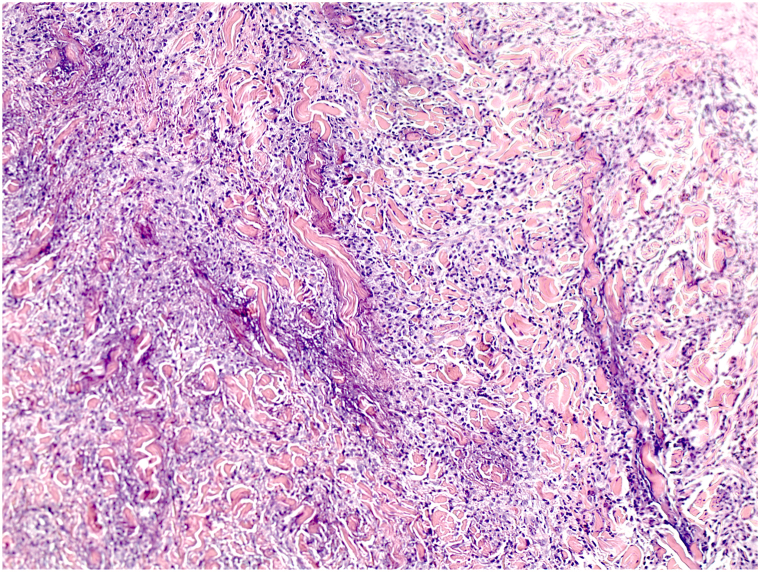
Detail of interstitial granulomatous infiltrate with degranulated neutrophils (×100, H&E).

Histology demonstrated sheets of CD163+/CD68+ histiocytes extending interstitially, as well as degranulation of neutrophils leading to the basophilic granular material coating the degenerating collagen seen in the dermis (Figs 2 and 3). The findings supported a diagnosis of interstitial granulomatous dermatitis (IGD).

He was continued on his current dose of methotrexate. On subsequent follow-up review 1 week postbiopsy, the left axilla lesion had spontaneously improved, while the right lesion persisted. He was treated with topical betamethasone dipropionate 0.05% ointment with a plan for intralesional triamcinolone injections for any persistent areas.

**Question: Interstitial granulomatous dermatitis is most commonly associated with which of the following systemic conditions?**

A. Systemic lupus erythematosus
B. Rheumatoid arthritis
C. Sarcoidosis
D. Inflammatory bowel disease
E. Granulomatosis with polyangiitis

## Discussion

The correct answer is option B – rheumatoid arthritis. A systematic review of systemic diseases associated with reactive granulomatous dermatitis including IGD reported that 47.6% of the 216 cases were associated with autoimmune disorders, most commonly rheumatoid arthritis (23.6%), followed by systemic lupus erythematosus (9.3%).^1^ Other associated conditions in the literature include lymphoproliferative disorders and certain medications such as tumor necrosis factor alpha inhibitors, calcium channel blockers, and 3-hydroxy-3-methylglutaryl-coenzyme A reductase inhibitors.^1^ The pathogenesis of IGD in autoimmune disease remains unclear. However, the strong association with autoimmune disease suggests a potential role for immune complex deposition.^2^ Specific IGD therapy is not well established, although reported treatment options including topical corticosteroids, methotrexate, phototherapy and oral glucocorticoids have been trialled.^3^^,^^4^ In this case, the patient demonstrated partial improvement with ongoing methotrexate therapy and topical steroids.

This case demonstrates the diagnostic significance of the rope sign as a distinctive but uncommon presentation of interstitial granulomatous dermatitis. Its identification should prompt clinicians to evaluate for associated systemic disease, particularly autoimmune conditions.

### Declaration of generative AI and AI-assisted technologies in the writing process

AI was not used in the preparation of this manuscript.

## Conflicts of interest

None disclosed.
